# Individual differences in training time in the rat gambling task are unrelated to subsequent decision-making strategies

**DOI:** 10.3389/fpsyt.2025.1490196

**Published:** 2025-01-27

**Authors:** Frida A. Lindberg, Christakis Kagios, Nikita Tjernström, Erika Roman

**Affiliations:** ^1^ Department of Animal Biosciences, Swedish University of Agricultural Sciences (SLU), Uppsala, Sweden; ^2^ Department of Medical Sciences, Uppsala University, Uppsala, Sweden; ^3^ Department of Pharmaceutical Biosciences, Uppsala University, Uppsala, Sweden

**Keywords:** behavioral profile, impulsivity, Iowa Gambling Task, learning speed, multivariate concentric square field, personality, risk taking behavioral profile, risk taking

## Abstract

**Introduction:**

Decision-making requires individuals to perceive probabilities and risks associated with different options. The Iowa gambling task (IGT) is a widely used instrument that assesses decision-making under uncertainty and risk by varying monetary reinforcer/loss contingencies. The rat gambling task (rGT), based on the IGT, is a preclinical test using varying number of palatable reinforcers as wins and different duration of timeouts as punishment, mimicking losses. The rGT requires extensive operant training prior to the free choice sessions. The aim of the present study was to investigate if task acquisition and number of training days affected subsequent individual differences in decision-making strategies in the rGT, and if behavioral profiles impacted on task learning.

**Method:**

Training time and performance of 70 male Lister Hooded rats from previously published studies were herein used to investigate whether learning time affected later decision-making strategies in the free choice rGT. Behavioral profiles generated from a subset of animals were used to study the impact of underlying behavior on learning time.

**Results:**

There were differences in training days between fast, intermediate and slow learners. However, time required to acquire the rGT did not affect subsequent decision-making strategies in the free choice rGT. Finally, learning time was independent of underlying behavioral profiles.

**Discussion:**

In conclusion, neither decision-making strategies in the rGT nor behavioral profiles were correlated or differed between animals with different learning speed. This suggests that the large variation in training time between animals is unrelated to subsequent decision-making strategies during free choice rGT. Such information is valuable for researchers using the rGT.

## 1 Introduction

Decision-making plays an essential role in everyday life and requires assessment of short- and long-term outcomes of probabilities and risks associated with different options. Impaired decision-making can be defined as a tendency to make unwise or risky options and is a core problem in several psychiatric conditions, including substance use and gambling disorders (1–3), attention deficit hyperactivity disorder (4), and affective disorders (5, 6). Research on decision-making processes and its involvement in psychiatric conditions have increased the last two decades, and several tests for different aspects of decision-making have been developed.

The Iowa gambling task (IGT) was originally created to assess impaired decision-making among patients with damage to the ventromedial prefrontal cortex (7). It has since become a widely used instrument for assessment of human decision-making under uncertainty and risk in clinical and non-clinical samples (8). The participants are presented with four decks of cards with different possibilities of winning or losing money. Unknown to the participants, the cards differ in their monetary gain/loss contingencies with two decks being advantageous and two decks being disadvantageous with regard to long-term monetary profit (7).

Several operant tasks are available for preclinical studies of different cognitive processes and underlying neurobiology, including delay discounting, five-choice serial reaction time task (5-CSRTT) and different versions of rodent gambling tasks. Importantly from a translational value, these tasks have human analogs (9–11). A commonality for the tasks, sometimes put forth as a caveat, is the intensive training required to teach the animal to perform the task before any experimental manipulations can take place. This makes them both time consuming and monetary costly (12). Moreover, training may bias the experimental results.

The rat gambling task (rGT) is based on the IGT and includes four choices associated with different probabilities of winning sucrose pellets or receiving punishing timeouts (13). To establish the most advantageous strategy, the rats need to prefer the low-risk options associated with small immediate rewards and short timeouts and avoid the options associated with larger immediate rewards and longer punishing timeouts. It has been shown that rats develop similar strategies in the rGT as humans in the IGT (14, 15), and the majority of rats learn and maintain a stable choice on the most advantageous option (13, 15–20). However, there are large individual differences (19, 20) and based on such individual strategies animals have been divided into three different strategy groups: (i) the strategic group, which prefers the most advantageous option, (ii) the safe group that prefers the safest option which gives one sucrose pellet and few and short timeouts, and (iii) the risky group with a higher preference for the disadvantageous options (19, 20).

There is a large variation in how long training is required for the rats to advance to the free choice rGT, but it is not known if task acquisition and number of training days differ between groups of rats with different decision-making strategies. It has previously been demonstrated that rats with different strategies in the rGT display differences in brain connectivity in regions associated with reward and decision-making processes (20). This finding implies underlying neurobiological differences that may impact on learning capability in the rGT. Rats with a risky strategy also displayed elevated motor impulsivity (19) but underlying risk-taking or shelter-seeking behavior did not explain individual differences in decision-making strategies in the rGT (20). However, it is not known if rGT task acquisition differs in rats with different learning speed or if there is an influence of the learning parameter that could impact on rGT results.

Data from two previous studies (19, 20) were herein combined and used to investigate whether individual decision-making strategies in the rGT are dependent on task learning acquisition, i.e. if the free choice rGT strategy differs between fast and slow learners. Moreover, behavioral profiles in a subset of animals (20) were used to explore if underlying behavior impacts on subsequent rGT task acquisition.

## 2 Materials and methods

### 2.1 Animals and housing

All animal experiments were approved by the Uppsala Animal Ethical Committee (permit number 5.8.18-00833/2017) and followed the guidelines of the Swedish Legislation on Animal Experimentation (Animal Welfare Act SFS 1998:56 and Animal Welfare Act SFS 2018:1192) and the European Union Directive on the Protection of Animals Used for Scientific Purposes (Directive 2010/63/EU).

Data used herein are combined from two previous studies and experimental details are given elsewhere (19, 20). Each rat was used in only one of the cohorts. Due to the larger prevalence of gambling problems in men than in women (23, 24), male rats were chosen. Part of the dataset has been used in a previous experiment for comparison with human decision-making strategies in the IGT (15).

In brief, a total of 72 Lister Hooded male rats (HsdOla: LH, Envigo, Horst, the Netherlands) were used; 32 rats in Tjernström et al. (20) and 40 rats in Tjernström and Roman (19). Animals were housed in pairs in transparent cages of type IV (dimensions 59 × 38 ×20 cm) with raised cage lids and wood chip bedding. All cages had bedding, two paper sheets (40 × 60 cm, Cellstoff, Papyrus) and a wood tunnel as enrichment. The housing room was operated on a reversed light/dark cycle (lights off at 06.00), with masking background noise, constant temperature (22 ± 1 °C) and humidity (50 ± 10%). All testing was performed during the dark phase of the light/dark cycle.

The animals were between five to six weeks old when delivered. After arrival, the animals were left undisturbed for two weeks to acclimate to the new environment and the reversed light/dark cycle. After acclimatization, the rats were handled for one week to get used to the experimenters and the weighing procedure.

During training and free choice rGT, the food was restricted to 85% of the free feeding weight to motivate the animals to perform in the operant boxes. The rats received 14 g of rat chow (type R36, Lantmännen, Kimstad, Sweden) one hour after their operant session. The rats had access to water *ad libitum*.

### 2.2 The rat gambling task

The procedure of the rat gambling task (rGT) is described in detail elsewhere (19, 20). Briefly, the rGT took place in five-hole operant chambers placed inside ventilated sound-attenuating cabinets (Med Associates, Inc.). The middle response hole was not used in either training or testing. The chambers had a house light and a food tray connected to a pellet dispenser. The rewards consisted of sucrose pellets (45 mg; Sandown Scientific, Middlesex, UK). Stimulus light and photo beams were mounted to the response holes and the food tray so that a record of responses could be made. The whole process was controlled by a computer software written in Med PC (Med Associates, Inc.). The operant chambers were cleaned with 10% ethanol after each session. The animals were then transferred back to their home cages after being weighed.

The task started with two daily 30-minute habituation sessions where sucrose pellets were placed in all response holes and in the food tray. Thereafter the training period started, which is described below.

#### 2.2.1 Training

Rats from both cohorts were trained according to the same protocol. Training was performed in one daily 30-minute session, for five consecutive days per week. The rGT training program consisted of six levels with each level increasing in difficulty, and the rats advanced to the next level by reaching a set goal. The rats were placed in the operant chambers and the session was initiated by the experimenter. For the trial to start, the rats had to nose poke in the illuminated food tray. Then, in the first level of training, the four response holes were illuminated and a response in any of them gave a reward of one sucrose pellet. The stimulus light was on until a response was made. The session lasted for 30 minutes or until the rats reached 100 completed trials. The first level was accomplished when 100 trials were completed under 30 minutes. At level two, only one of the response holes was illuminated and the rats were rewarded with one sucrose pellet when choosing that option. From level two and onwards, the duration of the stimulus light and the time to respond was gradually decreased for each level when the number of correct responses ≥ 80% and omissions ≤ 20%, eventually reaching 2 seconds.

#### 2.2.2 Forced and free choice rGT

**Figure 1:**
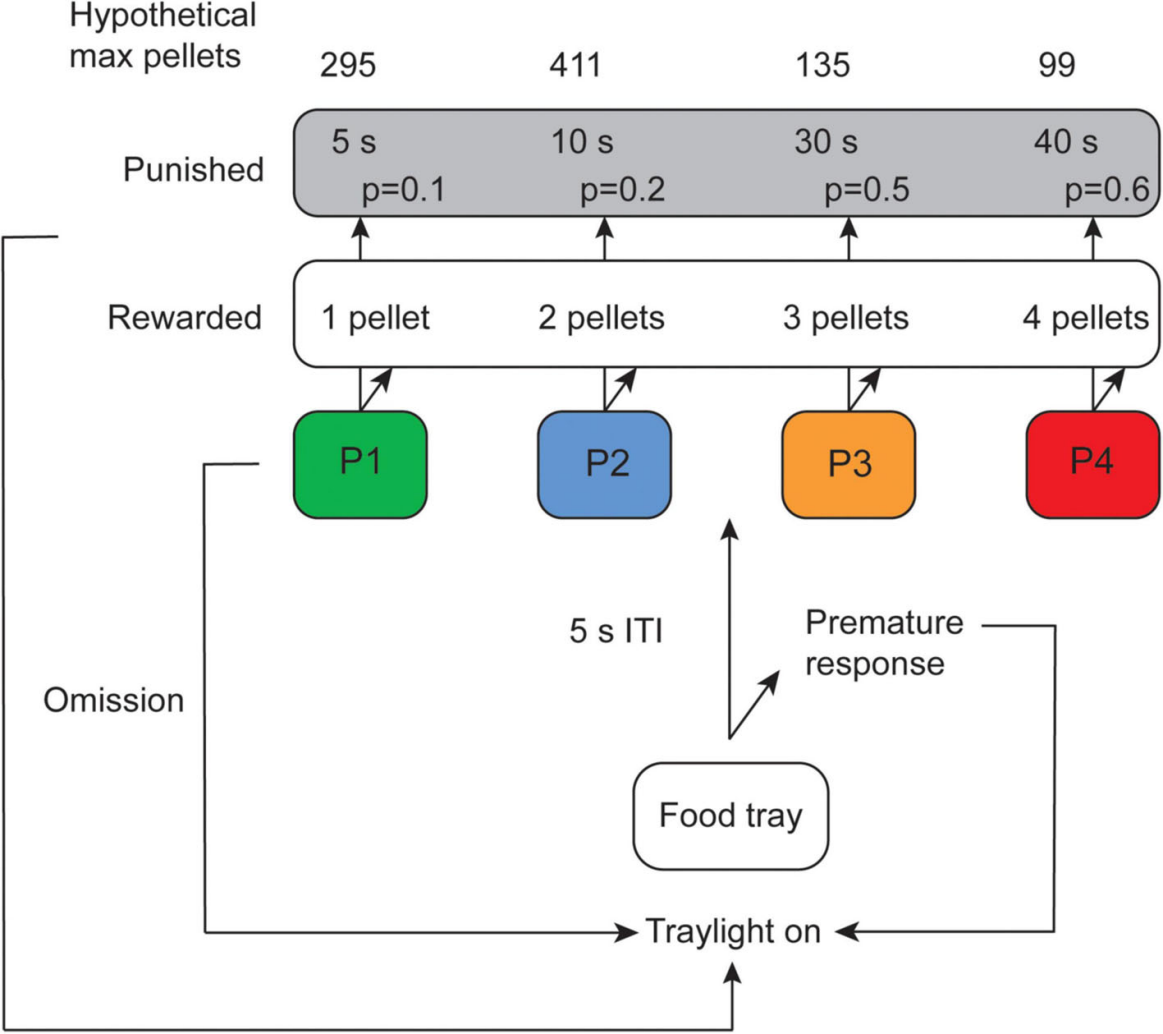
Schematic outline of the rat gambling task (rGT) showing the hypothetical maximum number of sugar pellets that could be earned for each choice during the 30 min session, duration and probability of punishing timeouts, and the number of rewarding pellets for each choice. ITI, inter-trial-interval. Illustration from Hultman et al. (15).

The rats had to initiate the session by nose poking in the illuminated food tray. In the last training level, the forced choice rGT, only one of the response holes was illuminated and led to either a reward or a punishment, with the same reinforcement schedule as the free choice rGT (**Figure 1**). This level was available for seven sessions to ensure that the rats were familiar with all the options (response holes) available.

In the free choice rGT, the rats had to wait for a 5 second inter trial interval (ITI) before the four response holes illuminated (**Figure 1**). Any response made during the ITI period was registered as a premature response (PR). When the response holes illuminated, the subjects were free to make a choice. In case they did not respond within 10 seconds, the trial was registered as an omission and the process started over. A nose poke in any of the four response holes, P1, P2, P3 and P4, was associated with different amounts of sucrose pellets or different length of timeout as punishment. Punishment probabilities, number of rewarding sugar pellets and duration of the punishing timeouts for each choice are summarized in **Figure 1**. A trial was considered completed when it ended with a reward or a punishment. Trials that ended in omissions or premature responses were not considered completed trials.

### 2.3 The multivariate concentric square field™ test

A detailed description of the multivariate concentric square field™ (MCSF) test can be found elsewhere (20, 25, 26). Briefly, the MCSF arena is 100 × 100 cm surrounded by walls (**Supplementary Figure S1**). The arena is divided into different zones, i.e. a central open area with a central circle allowing studies of center activity versus thigmotaxis, surrounding transit corridors, a dark closed (corner) room for shelter seeking, an elevated hole board area as explorative incentive, areas associated with risk assessment and a brightly illuminated bridge construction for studies of risk taking (**Supplementary Figure S1**). The animal is allowed to freely explore the different zones during a 20-minute trial and latency to first visiting, frequency of visits and total and mean duration of visits to the different zones are tracked. In addition, total distance and mean velocity in the different zones and in the arena are automatically tracked. Finally, the frequency of rearing, grooming, nose-pokes in the hole board holes and stretched attend postures are manually scored. The test is designed to assess a broader behavioral repertoire by collecting descriptive parameters of relevance to general activity, exploration, risk assessment, risk taking and shelter seeking (25–27).

To correlate learning time in the rGT to behavioral profiles in the MCSF test, the trend analysis from our previous study (20) was used. In the trend analysis, descriptive parameters within the same functional context are grouped together, ranked and summed into a sum rank for each functional category, i.e. general activity, exploratory activity, risk assessment, risk taking and shelter seeking, and compared in order to reveal group differences in behavioral profiles (20, 28).

### 2.4 Formation of strategy groups and statistics

Strategy groups were formed based on the average individual strategies from week five of the free choice rGT (19, 20). Week five was chosen because it included most of the rats (32 rats in (20) and 38 rats in (19); total n = 70) and the most reliable data since the animals had had time to stabilize their choices. Two of the rats (19) did not progress in the training phase to reach the free choice rGT, hence no data were available to include for analysis. Using the combined dataset, the different strategy groups were calculated as previously described (19, 20). The safe (n = 14) and strategic (n = 18) groups were formed from the upper quartile of P1 and P2, respectively. The risky group contained rats with P3% and P4% > Q3 + 1.5 × IQR (interquartile range; n = 14). The rest of the rats formed the group named other (n = 19). Five animals were not categorized since they belonged to two strategy groups according to the above criteria. Hence, the final number of animals used in the analyses was 65.

The rats were also categorized depending on how fast they were advancing through the rGT training. Using a tertiary split based on the number of rGT training days, the three groups fast (n = 24), intermediate (n = 24) or slow (n = 22) learners were formed.

The MCSF trend analysis, for a subset of the animals, was generated in a previous study (20). Descriptive parameters were grouped based on the functional categories general activity (total activity, number of visits to the corridors, duration per visit to the corridors [reversed], number of visits to the center and the total distance moved in the arena), exploratory activity (duration in the corridors [reversed], duration in the center [reversed], duration in the hurdle, number of rearings and nose-pokes in the hole board holes), risk assessment (stretched attend postures in the center and number of visits to, duration in and duration per visit in the slope), risk-taking (number of visits to, duration on and duration per visit on the bridge and number of visits to, duration in and duration per visit to the central circle), and shelter seeking (number of visits to, duration in and duration per visit to the dark corner room). Each parameter was ranked, so that the animal with the lowest score was given the lowest rank. Ranks belonging to the same functional category were summed up to a sum rank score. The rank sums were herein used to compare behavioral profiles in fast (n=9), intermediate (n=12) and slow (n=11) learners.

Statistical analyses were carried out in Statistica 13 (TIBCO Software Inc., Tulsa, OK, United States). Non-parametric statistics were used since most parameters were not normally distributed. The Kruskal-Wallis test was used to examine significant differences among more than two groups followed by *post-hoc* analysis and Bonferroni multiple correction where appropriate. Tests were considered significant at adjusted p < 0.05. Spearman rank order correlation was used to investigate associations between learning time and behavioral profiles in the MCSF trend analysis, as well as between learning time and rGT parameters. Correlation analyses were considered significant at p < 0.004 (Bonferroni correction for 13 comparisons). All figures were created with GraphPad Prism (Version 10.2.1, GraphPad Software, San Diego, CA, United States).

Additionally, SIMCA 17 (Sartorius Stedim Data Analytics AB, Umeå, Sweden) was used for the principal component analysis (PCA) to illustrate individual rats and loading of individuals according to decision-making strategies.

## 3 Results

**Figure 2:**
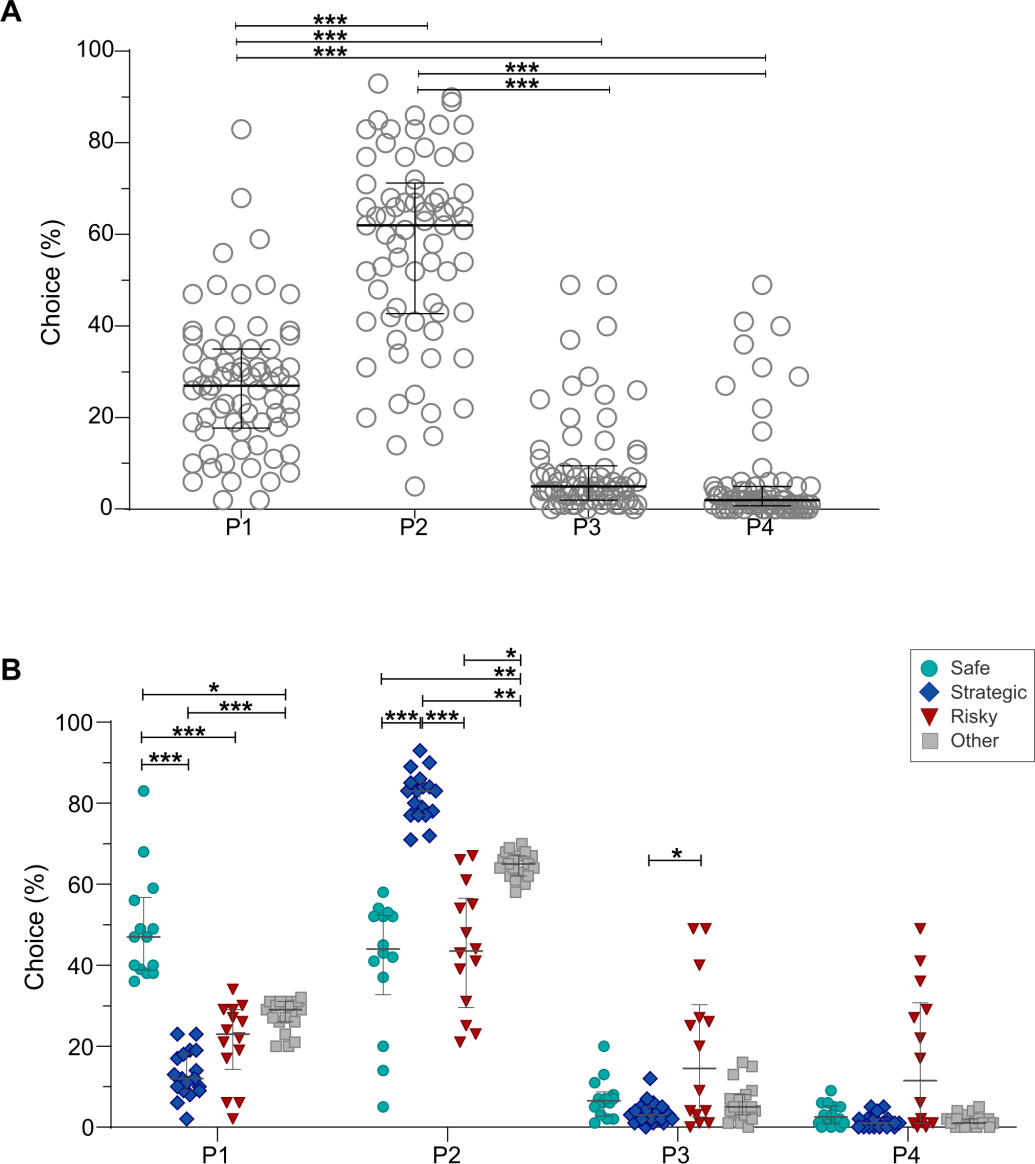
Preferred choices in all rats (n = 70) **(A)**, and in the strategy groups safe (n = 14), strategic (n = 18), risky (n =14), and other (n = 19) **(B)** in the free choice rGT. Data are shown as individual animals with median and interquartile range. *p < 0.05, **p < 0.01 and ***p < 0.001 (Kruskal-Wallis test with *post-hoc* Bonferroni correction).

On a population level, the rats required on average 21.6 days (median 22.5, range 11–34) of training to reach the free choice rGT. As expected, the free choice rGT results for all rats in the combined dataset showed that P2 was the most preferred choice over all other choices (p < 0.001), followed by P1 (p < 0.001). The disadvantageous choices P3 and P4 did not differ (**Figure 2A**). Ratios of completed trials, omissions and premature responses are shown in **Supplementary Figures S2A–C**, revealing large variation in ratio for premature responses.

The individual rGT choices in the different strategy groups revealed that the rats in the safe group preferred the choice P1 significantly more compared to all other groups (strategic p < 0.001; risky p < 0.001; other p < 0.05). Moreover, the strategic rats had a lower preference for P1 (p < 0.001) compared to rats in the group other. The strategic rats had a higher preference for the choice P2 compared to all other groups (safe p < 0.001; risky p < 0.001; other p < 0.01). Moreover, the safe and risky rats had a lower preference for P2 (p < 0.01 and p < 0.05, respectively) compared to rats in the group other. For P3, the risky group had a higher preference than the strategic group (p < 0.05; **Figure 2B**). No difference in the choice of P4 was found between the groups, although a trend was found between the strategic and risky rats (p = 0.054). Regarding ratios, risky rats were found to have a higher number of completed trials than all other strategy groups (safe p < 0.05; strategic and other p < 0.01), as well as a lower ratio of omissions compared to safe, strategic and the group other (p < 0.05, p < 0.01 and p < 0.01, respectively; **Supplementary Figures S2D, E**), while no differences were found for premature responses (**Supplementary Figure S2F**). The grouping of rats into safe, strategic, and risky based on statistical calculations for each choice was further supported by the PCA score plot (**Supplementary Figure S3A**), which revealed a clear separation between the individuals belonging to the different strategies based on choices (P1–P4; **Supplementary Figure S3B**).

No differences in number of training days needed to acquire the task were found between the safe, strategic, and risky groups (**Table 1**), and neither were there any correlations between learning time and any of the rGT choices (P1–4), number of omissions, completed trials, total trials or premature responses (**Supplementary Table S1**).

**Table 1:** Number of training days required for the strategy groups safe (n = 14), strategic (n = 18), risky (n =14), and other (n = 19) to reach the free choice rGT.

Strategy	Median	Quartile range	Min–max
Safe	23.5	21.3–26.0	11.0–26.0
Strategic	21.5	17.8–23.8	12.0–25.0
Risky	22.5	18.0–25.0	12.0–34.0
Other	23.0	17.5–25.0	14.0–31.0

**Figure 3:**
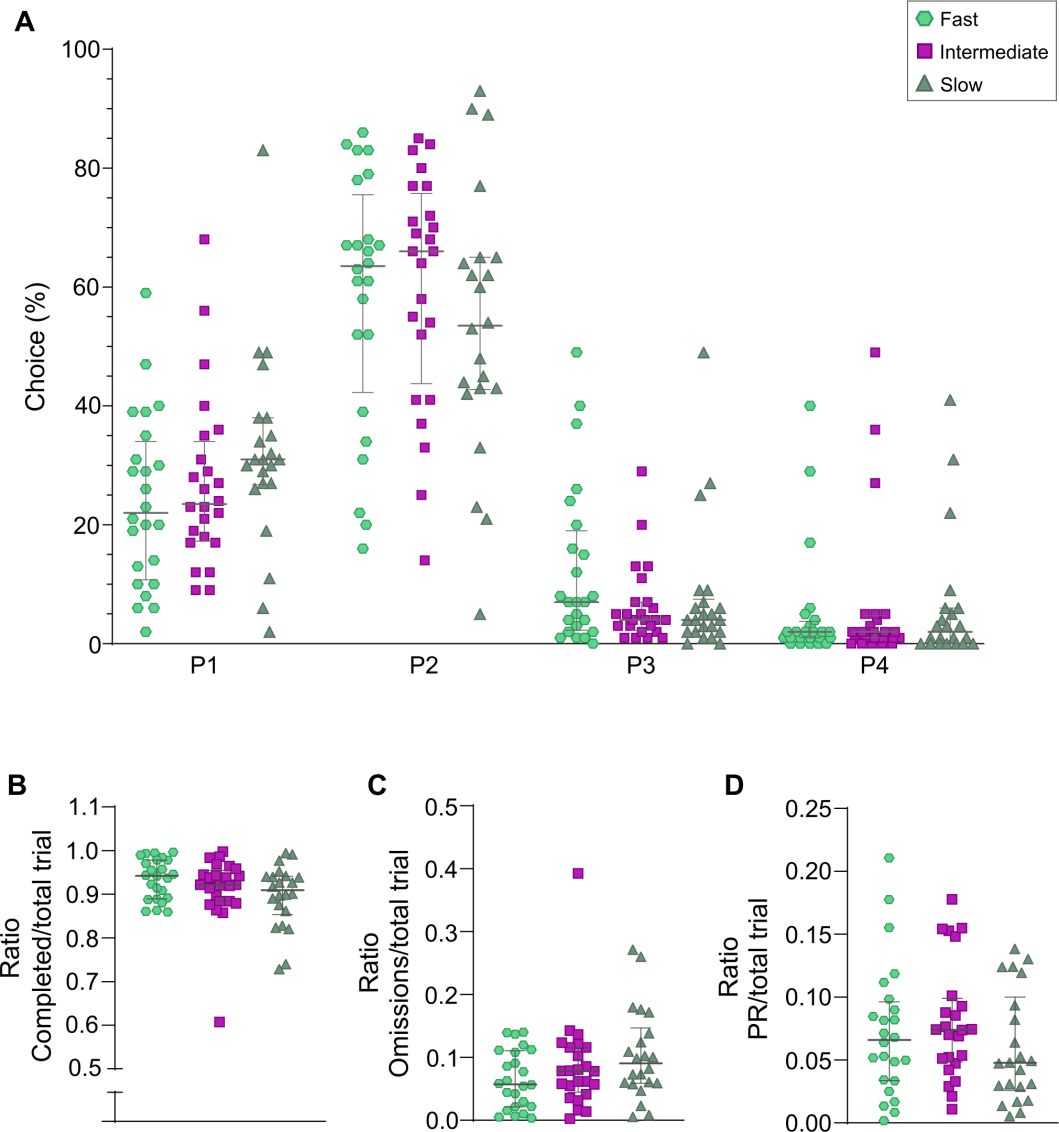
rGT results in rats categorized as fast (n = 24), intermediate (n = 24), or slow (n = 22) learners based on number of rGT training days, showing preferred choices **(A)**, as well as ratios of completed trials **(B)**, omissions **(C)**, and premature responses (PRs; **D**) against total trials. Data are shown as individual animals with median and interquartile range. No significant differences between the groups were revealed (Kruskal-Wallis test).

When animals were grouped into fast, intermediate or slow learners based on the number of rGT training days (**Table 2**), significant differences were revealed between fast and slow learners (p < 0.001), fast and intermediate learners (p < 0.05) and intermediate and slow learners (p < 0.01). The preferred choice in the rGT did not differ between animals with different learning speed (**Figure 3A**), and neither did the ratios of completed trials, omissions or premature responses (**Figures 3B–D**).

**Table 2:** Number of training days required to reach the free choice rGT in fast (n = 24), intermediate (n = 24) and slow (n = 22) learners.

Learning time	Median	Quartile range	Min–max
Fast ^a,b^	16.0	14.8–18.3	11.0–20.0
Intermediate	23.0	22.0–23.0	21.0–24.0
Slow ^c^	26.0	25.0–26.0	25.0–34.0

^a^ p < 0.05 compared to intermediate learners, ^b^ p < 0.001 compared to slow learners, and ^c^ p< 0.01 compared to intermediate learners.

**Figure 4:**
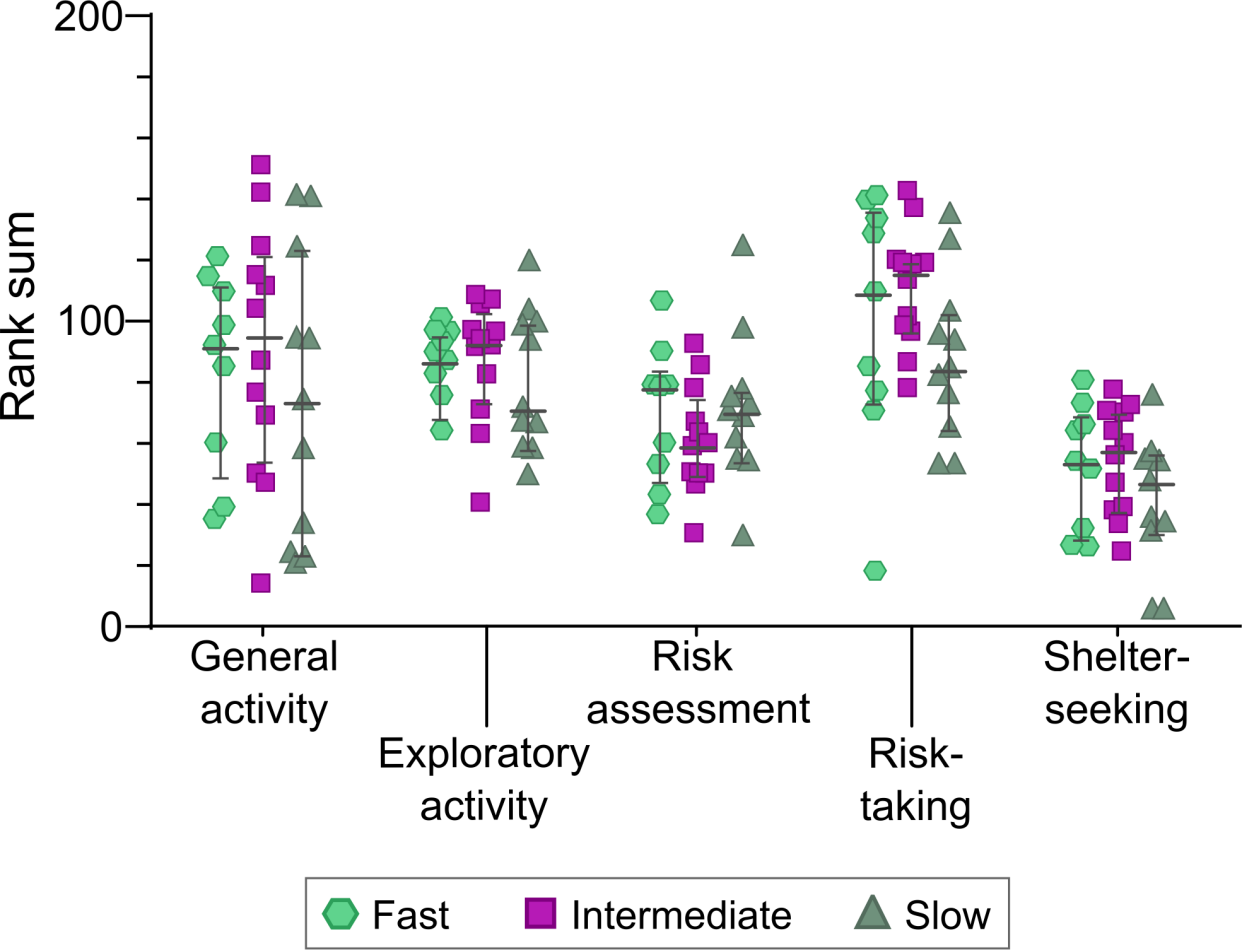
Results from the MCSF trend analysis generated in Tjernström et al. (20) in rats later categorized as fast (n = 9), intermediate (n = 12) or slow (n = 11) learners based on number of rGT training days. Data are shown as individual animals with median and interquartile range. No significant differences between the groups were revealed (Kruskal-Wallis test).

No differences in underlying behavioral profiles were found between fast, intermediate and slow learners in the rGT (**Figure 4**). Finally, no correlations between MCSF performance and number of training days were found (**Supplementary Table S2**).

## 4 Discussion

In the current study, datasets from two previous experiments (19, 20) were combined in order to increase the number of animals and thereby the statistical power. The results revealed large individual differences in the number of training days required to reach the free choice rGT, but that number of training days was unrelated to later decision-making strategies in the rGT. Such information is useful from a methodological perspective. Moreover, in a subset of the animals it was also found that underlying behavioral profiles were unrelated to later training time required to reach the free choice rGT.

Operant tasks for assessment of complex cognitive function demand for multiple processes to be integrated, including attention, information acquisition, learning and memory, and require intense training in order for the animal to learn the task and advance to the next step (e.g. 12, 13, 21, 22). It is therefore not surprising to see large individual variation, as revealed herein. Research in the field of decision-making has often ignored individual differences (29). For example, studies using the rGT have mainly focused on experimental manipulations and treatments that attenuate the disadvantageous options in favor of the more advantageous choices (13, 16, 30–32). To the best of our knowledge, no study has reported individual differences in operant training time and if there are any correlations between learning speed and decision-making strategy.

Individual differences in cognitive function have been proposed to be dependent on the interplay between risk–reward trade-offs, cognitive styles and behavioral types (33) although also non-cognitive factors can cause variation in performance (34). The risk–reward trade-off (33) is evident in the rGT given the advantageous versus disadvantageous and riskier options. As revealed herein, large individual differences in free choice rGT performance were found and that was the basis for the formation of the strategy groups strategic, safe and risky in agreement with previous studies (19, 20). Previous work on a smaller subset of the rats used herein has demonstrated that the rGT strategies are stable over time, also with multiple interruptions in rGT testing (19). The risky decision-making strategy was associated with the lowest choice of advantageous options (P1 or P2) and highest preference for the disadvantageous options (P3 or P4). However, the risky rats still made choices from P2 suggesting that they might distinguish the most advantageous option but still went for the immediate larger rewards that are available with the risker options of P3 or P4. Furthermore, when correcting for the number of total trials, rats in the risky group had a higher ratio of completed trials and fewer omissions compared to the other groups. Omissions are often interpreted as lack of motivation (19, 35). Thus, rats with risky decision-making in the rGT may be characterized by higher motivation to gain rewards and to complete trials once initiated. This finding could indicate that risky rats are more reward sensitive, in agreement with findings using another version of the rGT (36). However, this finding could also indicate that risky rats are more insensitive to punishment. For such a conclusion to be drawn, the results herein need further investigation.

Regarding the interplay between risk–reward trade-offs and cognitive styles underlying individual differences in cognitive function (33), the speed at which an animal learns an operant task is a common measure of cognition (34). The rGT training is based on that in the 5-CSRTT, i.e. a demand for intense training (21). In previous studies, male Lister Hooded rats reached a stable performance in the 5-CSRTT after approximately 35–40 daily sessions (37, 38). However, information about range or individual differences was not provided (37, 38). There have been attempts to speed up learning, but training still takes several weeks (12). Herein the combined population of rats required on average 21.6 days of training (range 11–34) to reach the free choice rGT. There were no differences in learning time between the strategy groups. Since risk–reward trade-off and speed of learning may impact on later performance, this is valuable information for researchers using the rGT.

When the rats were divided into groups based on number of training days required, i.e. fast, intermediate and slow learners, no group differences in decision-making strategy were found. This is in line with findings from the 5-CSRTT, where no differences in task accuracy were found between fast and slow learners (12). However, slow learners had an increased percentage of omissions and a decreased number of premature responses compared to fast learners (12), which contrasts the findings herein where no group differences were revealed for neither omissions nor premature responses. Bhandari et al. (12) also observed a tendency to a positive correlation between learning time and percentage of omissions, although no such tendencies were seen in data analyzed herein. In accordance with Bhandari and colleagues (12) no correlation between premature responses and training time was found. Together these results suggest that free choice rGT performance is not affected by learning speed.

Individual differences in cognitive function have also been linked to the interplay between risk–reward trade-offs and animal personality (33). The fact that no differences in learning time were found when comparing the strategy groups nor when comparing animals grouped based on learning time, indicates that factors underlying operant learning are not the main contributors to later individual differences in decision-making strategies in the free choice rGT. In support of this is a recent meta-analysis that did not find a clear relationship between animal personality and learning. The direction of relationships differed between the included studies, resulting in an average effect size that did not differ from zero, and a major part of the variation between studies remained unexplained (39).

In the study by Hultman et al. (15), a comparison of the performance by humans in the IGT and rats in the rGT was performed. Both similarities and differences between the two tasks were discussed. Two important factors that differ between the two tasks are the training and the number of trials. In the IGT, 100 trials are carried out in one session without prior training, while rats in the rGT, after extensive training, performed 25 sessions in the free choice rGT. Hence, humans and rats are most probably relying on different memory systems when making their decisions. Despite these discrepancies, both human participants and rats showed similar subgroup formations at the end of the tasks (15), and the results herein suggest that the extensive training in rats do not impact on the strategies that are developed.

This study was based on performance in the rGT by male rats, hence conclusions regarding female performance and its correlation to task acquisition in the rGT is unknown. Since both decision-making (40–42) and learning (43, 44) have been reported to differ between males and females, the findings herein cannot be translated to females. Another limitation is the low number of animals in the analyses of behavioral profiles and learning, which makes it less powerful than remaining analyses. However, we show that the length of training in the rGT is independent of later decision-making strategies, which is useful knowledge when interpreting results from the rGT. To continue the study of cognitive parameters and learning in the rGT, an interesting task would be to identify the risky decision-makers and further investigate their learning ability, including selective attention. If the risky strategy group is more reward sensitive than others, an impaired selective attention would be anticipated (45).

In conclusion, operant learning time in the rGT was unrelated to later decision-making strategies. From a methodological perspective such information is valuable for researchers using the rGT.

## Data Availability

The raw data supporting the conclusions of this article will be made available by the authors, without undue reservation.
