# Supplementary material for: Individual differences in training time in the rat gambling task are unrelated to subsequent decision-making strategies
Source: Front Psychiatry. 2025 Jan 27;16:1490196. doi: 10.3389/fpsyt.2025.1490196 (PMC11808017; doi:10.3389/fpsyt.2025.1490196)
Supplement: Supplementary file 1 [file DataSheet1.docx]

Supplementary Figures


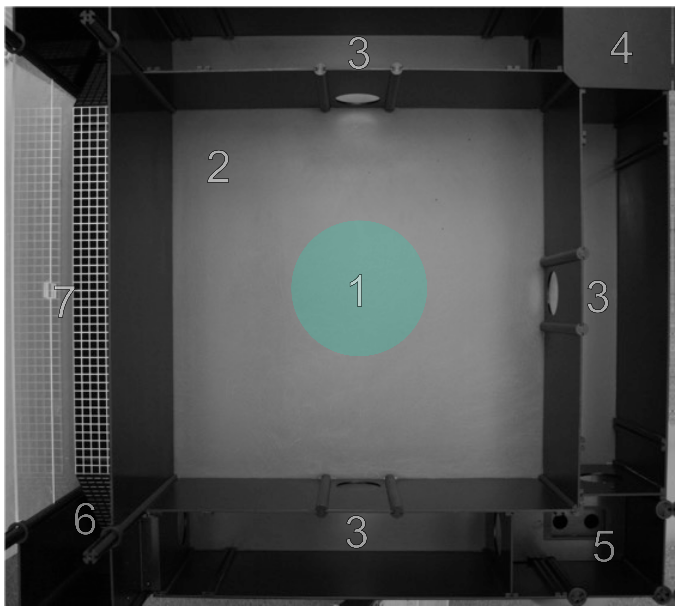


**Figure S1**. The multivariate concentric square field^TM^ (MCSF) test with the defined zones (1) central circle, (2) center, (3) corridors, (4) dark corner room, (5) hurdle with a hole board, (6) slope, and (7) bridge.


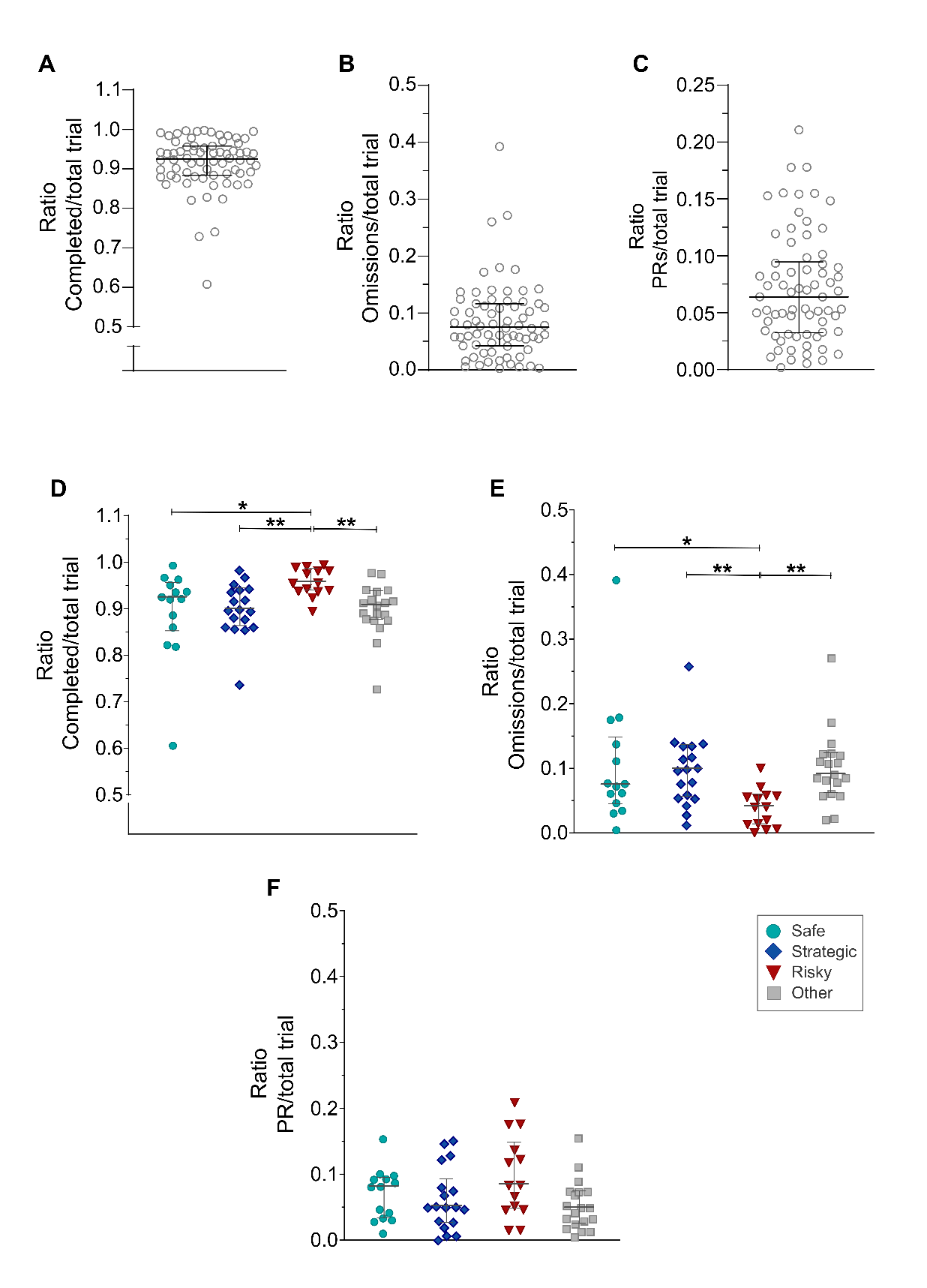


Figure S2. Ratio of completed trials (A), omissions (B), and premature responses (PRs; C), in all male rats in the rGT (n = 70), followed by the ratio of completed trials (D), omissions (E), and premature responses (PRs; F) ) in male rats with safe (n = 14), strategic (n = 18) and risky (n = 14) decision-making strategies in the rGT, as well as in rats belonging to the group other (n = 19). Data are shown as individual animals with median and interquartile range. *p < 0.05, **p < 0.01 (Kruskal-Wallis test with post-hoc Bonferroni correction).


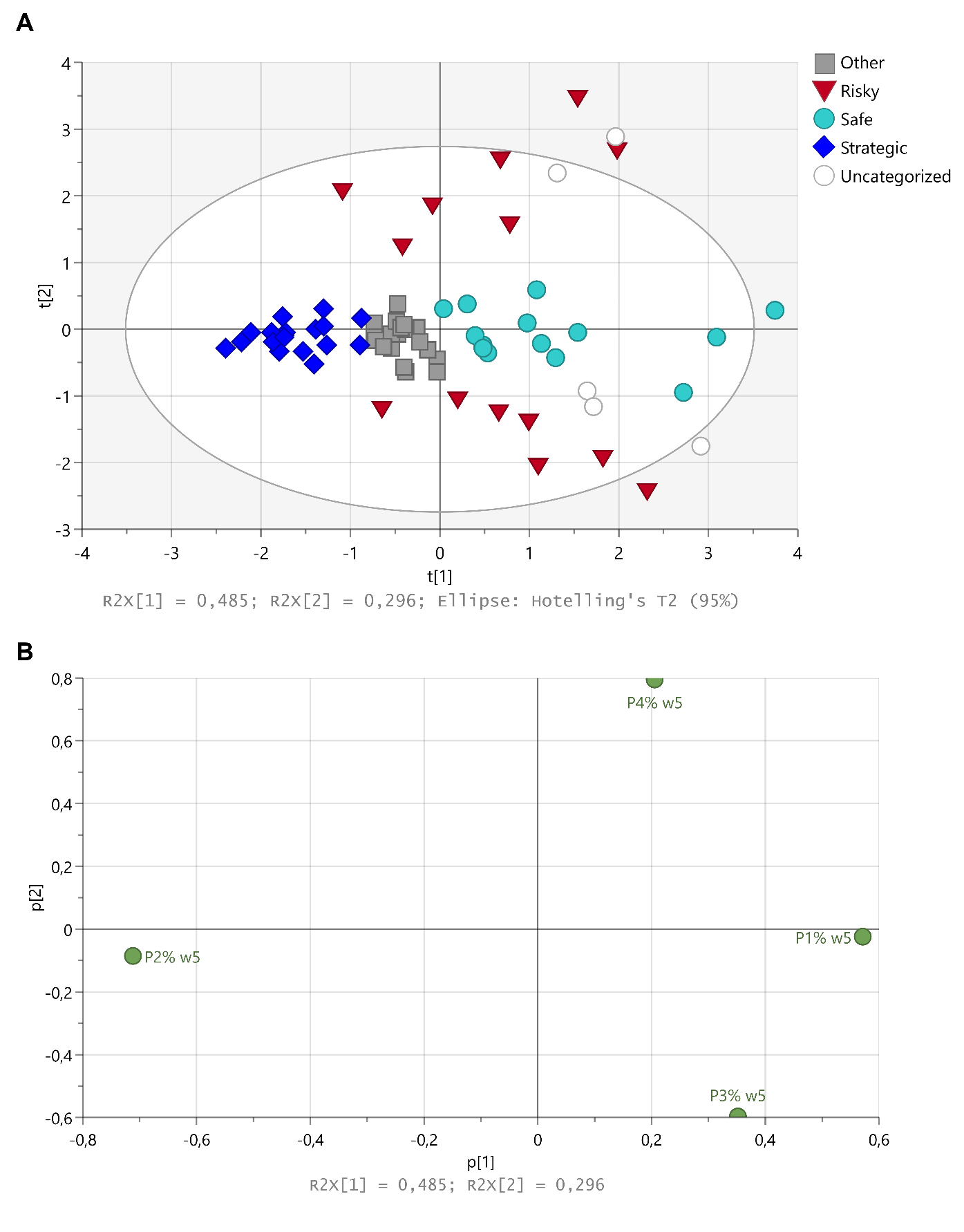


**Figure S3.** Principal component analysis (PCA) score plot (**A**) showing the individual animals and loading plot (**B**) showing the parameters of importance for the individual loadings. No significant components (R2X(cum) = 0.781, Q2(cum) = -0.046).

Supplementary Tables

**Table S1.** Summary of correlation analyses between number of rGT training days and different parameters measured in the rGT.

|  | Spearman R | p-value |
| --- | --- | --- |
| Training days – Omissions | 0.22 | 0.067 |
| Training days – Completed trials | -0.13 | 0.283 |
| Training days – Total trials | -0.04 | 0.753 |
| Training days – PRs | -0.13 | 0.271 |
| Training days – P1 | 0.27 | 0.023 |
| Training days – P2 | -0.13 | 0.304 |
| Training days – P3 | -0.19 | 0.116 |
| Training days – P4 | 0.03 | 0.837 |

Correlations were considered significant at p < 0.004 (Bonferroni correction). P1–4, percentage of choice for each option; PRs, premature responses.

**Table S2.** Summary of correlation analyses between number of rGT training days and MCSF trend analysis results from Tjernström *et al.* (2022).

|  | Spearman R | p-value |
| --- | --- | --- |
| Training days – TA General activity | -0.05 | 0.791 |
| Training days – TA Exploration | -0.03 | 0.861 |
| Training days – TA Risk assessment | -0.03 | 0.894 |
| Training days – TA Risk taking | -0.21 | 0.244 |
| Training days – TA Shelter seeking | -0.14 | 0.453 |

Correlations were considered significant at p < 0.004 (Bonferroni correction). TA, MCSF trend analysis.
